# Voriconazole therapy and *CYP2C19* phenotype: identifying patients who may need alternative antifungal therapy

**DOI:** 10.1093/jac/dkag168

**Published:** 2026-05-19

**Authors:** Eman Wehbe, Thulashigan Sreeharan, Gaurav Sutrave, Jessica Bui, Cindy Lau, Jung Yeun Park, Indy Sandaradura, David Darley, Peter Macdonald, Samuel Milliken, Jiin Nyuk Fung, Graham Jones, Alice Kizny Gordon, David Kliman, Christine Y Lu, Deborah Marriott, Jan-Willem C Alffenaar, Sophie L Stocker

**Affiliations:** School of Pharmacy, Faculty of Medicine and Health, The University of Sydney, Sydney, NSW 2006, Australia; Department of Pharmacy, Westmead Hospital, Sydney, NSW 2145, Australia; Department of Pharmacy, Westmead Hospital, Sydney, NSW 2145, Australia; Westmead Institute for Medical Research, University of Sydney, Sydney, NSW 2145, Australia; Department of Pharmacy, Royal North Shore Hospital, Sydney, NSW 2065, Australia; Department of Pharmacy, St Vincent’s Hospital, Sydney, NSW 2010, Australia; Department of Pharmacy, Westmead Hospital, Sydney, NSW 2145, Australia; Westmead Institute for Medical Research, University of Sydney, Sydney, NSW 2145, Australia; Centre for Infectious Diseases and Microbiology, Westmead Hospital, Sydney, NSW 2145, Australia; Sydney Institute for Infectious Diseases, The University of Sydney, Sydney, NSW 2145, Australia; Department of Thoracic Medicine, St Vincent’s Hospital, Sydney, NSW 2010, Australia; Heart and Lung Transplant Unit, St Vincent's Hospital, Sydney, NSW 2010, Australia; Department of Haematology, St Vincent’s Hospital, Sydney, NSW 2010, Australia; Department of Pharmacy, Westmead Hospital, Sydney, NSW 2145, Australia; Department of Chemical Pathology and Clinical Pharmacology, SydPath, St Vincent's Hospital, Sydney, NSW 2010, Australia; Department of Infectious Diseases and Microbiology, Royal North Shore Hospital, Sydney, NSW 2065, Australia; Department of Haematology, Royal North Shore Hospital, Sydney, NSW 2065, Australia; School of Pharmacy, Faculty of Medicine and Health, The University of Sydney, Sydney, NSW 2006, Australia; Department of Pharmacy, Royal North Shore Hospital, Sydney, NSW 2065, Australia; Kolling Institute, Faculty of Medicine and Health, The University of Sydney, The Northern Sydney Local Health District, Sydney, NSW 2065, Australia; Department of Clinical Microbiology and Infectious Diseases, St Vincent’s Hospital, Sydney, NSW 2010, Australia; School of Pharmacy, Faculty of Medicine and Health, The University of Sydney, Sydney, NSW 2006, Australia; Department of Pharmacy, Westmead Hospital, Sydney, NSW 2145, Australia; Sydney Institute for Infectious Diseases, The University of Sydney, Sydney, NSW 2145, Australia; School of Pharmacy, Faculty of Medicine and Health, The University of Sydney, Sydney, NSW 2006, Australia; Department of Pharmacy, Westmead Hospital, Sydney, NSW 2145, Australia; Sydney Institute for Infectious Diseases, The University of Sydney, Sydney, NSW 2145, Australia; Department of Clinical Pharmacology and Toxicology, St Vincent’s Hospital, Sydney, NSW 2010, Australia

## Abstract

**Background and objectives:**

*CYP2C19* phenotype is a known contributor to the interpatient variability in voriconazole response. Alternative therapy is recommended for ultrarapid/rapid and poor metabolizers due to increased probability of subtherapeutic exposure and side effects, respectively. We aimed to evaluate whether *CYP2C19* phenotype is associated with switching from voriconazole to alternative antifungal therapy, in settings where genetic results were not available at the time prescribing decisions were made.

**Methods:**

A multicentre, retrospective observational study was conducted in three Australian hospitals. Patients who had previously taken voriconazole (from 1 May 2019 to 31 May 2024) were invited to undergo pharmacogenomic testing. Medical records were audited to compare switching decisions across patients with different *CYP2C19* phenotypes. Differences in voriconazole exposure and voriconazole-related adverse effects were also explored.

**Results:**

Among 194 patients, most were normal or intermediate metabolizers (69%); 21% were rapid, 7% ultrarapid, and 3% poor metabolizers (underpowered). Switching to alternative antifungal therapy (32%; 62/194) mainly occurred due to adverse effect incidence and was not associated with *CYP2C19* phenotype (*P* = 0.9041). C-reactive protein levels were significantly higher in patients who switched therapy (*P* < 0.001). All ultrarapid metabolizers on standard voriconazole 400 mg/day had subtherapeutic concentrations and only those on higher doses (500–1200 mg/day) achieved therapeutic concentrations.

**Conclusions:**

*CYP2C19* phenotype was not predictive of switching, which is a multifactorial prescribing decision likely influenced by therapeutic drug monitoring, inflammation and clinical status. Our observations on voriconazole dosing and exposure support a complementary prescribing approach: pharmacogenomic testing to identify patients who require atypical voriconazole dosing regimens and subsequent therapeutic drug monitoring to guide dose adjustments.

## Introduction

Invasive fungal infections, particularly aspergillosis, remain a major complication in transplant recipients, occurring in 23%–26% of solid organ and up to 40% of haematopoietic stem cell transplant recipients,^1–3^ with 12 week mortality rates of 38%–79%.^1–4^ This underscores the need for timely initiation of targeted and effective antifungal therapy.^4^ Voriconazole is one of the first-line agents for treatment and prophylaxis of invasive aspergillosis in this patient population.^1,5^ However, voriconazole exhibits highly variable pharmacokinetics, with 36%–45% of patients not achieving therapeutic concentrations at recommended doses (4 mg/kg or 200 mg twice daily).^6–9^ This contributes to significant rates of treatment failure (25%–64%) and adverse effects (40%–60%).^10–14^ The narrow therapeutic range (1.0–5.5 mg/L), saturable metabolism, and marked (>70%) interpatient variability in exposure of voriconazole complicate optimal administration.^7,15,16^ Therefore, discontinuation of voriconazole and the use of alternative antifungals is common.

One major contributor to the variability in the pharmacokinetics of voriconazole is genetic variation in cytochrome P450 2C19 (*CYP2C19),* which is responsible for metabolizing the drug in the liver.^17,18^ Up to 49% of the variability in drug exposure can be explained by *CYP2C19* genotype^19^ and corresponding ‘phenotypes’ based on metabolic activity (Table 1). Patients classified as ultrarapid or rapid metabolizers clear voriconazole more quickly, leading to lower plasma drug concentrations and increased risk of subtherapeutic exposure.^21,22^  *CYP2C19* ultrarapid or rapid metabolizers have a higher prevalence of subtherapeutic voriconazole trough concentrations than those with other *CYP2C19* phenotypes.^22^ This leads to increased risk of disease progression.^23^ Conversely, poor metabolizers have reduced clearance, resulting in elevated voriconazole concentrations and a higher risk of adverse effects.^14,24–26^ In 2020, the Clinical Pharmacogenetics Implementation Consortium (CPIC) issued guidelines recommending the use of alternative antifungals for patients with ultrarapid, rapid and poor *CYP2C19* metabolizer phenotypes,^20^ as standard voriconazole dosing may not be appropriate for these patients.

**Table 1.** *CYP2C19* genotype and the associated phenotype^20^

*CYP2C19* allele combinations (genotype)		Enzyme function (phenotype)
*1/*1	2 normal function alleles	Normal metabolizer
*17/*17	2 increased function alleles	Ultrarapid metabolizer
*1/*17	1 normal + 1 increased function allele	Rapid metabolizer
*2/*2, *3/*3	2 no function alleles	Poor metabolizer
*1/*2, *1/*3, *2/*17	1 normal + 1 no function allele OR 1 increased + 1 no function allele	Intermediate metabolizer

This retrospective study aimed to determine whether *CYP2C19* phenotype is associated with switching from voriconazole to an alternative antifungal in settings where pharmacogenomic results were not available at the time of prescribing. Switching outcomes were examined among patients who later consented to *CYP2C19* testing to assess the potential value of pharmacogenomic testing in the optimization of antifungal selection and prescribing. Secondary aims included assessing associations between *CYP2C19* phenotype, voriconazole exposure and the occurrence of voriconazole-related adverse effects.

## Methods

### Study design

A multicentre, retrospective cohort study was conducted at three tertiary hospitals in Sydney, Australia: Westmead Hospital, Royal North Shore Hospital and St Vincent’s Hospital. Adults (aged ≥18 years) who received voriconazole between 1 May 2019 and 31 May 2024, and had at least one voriconazole plasma concentration recorded, were invited to participate. Written informed consent was obtained for *CYP2C19* genotyping. Patients who received voriconazole for less than 5 days were excluded as this duration is generally required to approach pharmacokinetic steady state and allow meaningful interpretation of exposure. Ethics approval was granted by the Western Sydney Local Health District Ethics Committee (2022/ETH00951).

### Data collection

#### Clinical data

Clinical data were collected from the medical records including baseline patient characteristics, details of voriconazole therapy (dose, route of administration, duration), voriconazole-related adverse effects, details of alternative antifungal treatment (where applicable) and the reason for switching to an alternative antifungal agent as noted by the prescribing physician. Pathology data (serum creatinine, liver function biomarkers, C-reactive protein)^27,28^ at the start and end of voriconazole therapy were also collected. Voriconazole dose was recorded as milligrams per day. Where more than one voriconazole dosing regimen and route of administration was used, the final dose and route of administration was recorded. If a patient experienced an adverse effect, the dosing regimen and route of administration at the time was also recorded.

Cases of invasive aspergillosis were classified according to the European Organisation for Research and Treatment of Cancer and Mycoses Study Group definitions.^29^ Adverse effects were included if ascribed to voriconazole in clinical notes and classified into four categories: (i) Hepatotoxicity—included derangement in liver function biomarkers; (ii) Skin reaction—included rashes, phototoxicity; (iii) Neurotoxicity—included vivid dreams, visual disturbances, dizziness, confusion, hallucinations, irritation; (iv) Other—included QTc prolongation, nausea, muscular pain.

Patients administered an alternative antifungal agent instead of or in addition to voriconazole were referred to as ‘switched’. Patients who remained on voriconazole antifungal monotherapy were referred to as ‘non-switched’. Ethnicity was self-reported using the Australian Standard Classification of Cultural and Ethnic Groups.^30^

#### Estimation of voriconazole exposure

Voriconazole exposure was modelled using the Insight RX Nova Bayesian dosing platform (Version 1.36.6, Insight Rx Inc., 2021). Inputs included demographic data (age, sex, weight, height), voriconazole dosing information (dose, administration date and time), and observed plasma voriconazole concentrations. Bayesian pharmacokinetic modelling^31^ was used, with maximum a posteriori estimation and flattened priors. All observed values were given equal weighting in the model. For concentration–time profiles with an intermediate or poor model fit, re-analysis using least-squares fitting methods was conducted. For each patient, the average trough concentration and AUC_0–24_ were calculated for the final voriconazole dosing regimen. For patients who experienced a drug-related adverse effect, average trough concentrations and AUC_0–24_ values were based on the dosing regimen administered at the time of the event. Trough voriconazole concentrations between 1.0 and 5.5 mg/L were classified as therapeutic.^15,16^

#### CYP2C19 genotyping

Genotyping was performed using buccal swabs, self-collected via mailed DNA test kits (myDNA^TM^) or obtained during clinic visits. Genotyping was conducted using TaqMan based assays for CYP2C19*2, *3 and *17. Phenotype classifications were assigned based on the Pharmacogene Variation Consortium and the Clinical Pharmacogenetics Implementation Consortium (Table 1).^20,32^ An interpretative pharmacogenomic report was generated for each patient and shared with clinicians and patients after study completion.

### Statistical analysis

Descriptive statistics were used to summarize patient and treatment characteristics. Between-group comparisons (switched versus non-switched) were conducted using the chi-squared test or Fisher’s exact test for categorical variables, and *t*-tests or Mann–Whitney *U* for continuous variables. The relationship between *CYP2C19* phenotype and switching to alternative therapy was assessed using a logistic regression model adjusted for identified covariates. Associations between *CYP2C19* phenotype and voriconazole trough concentrations or drug-related adverse effects were evaluated using the Kruskal–Wallis and chi-square tests, respectively. Sensitivity analysis, including measurement of AUC, were performed to confirm associations with trough concentrations.

No patients were excluded from the study due to missing data. Patients were excluded from specific analyses if they had missing data for any variable required in that analysis, using a listwise deletion approach. Data analysis was performed using Jamovi (version 2.6.44) and GraphPad Prism (version 9.5.1). A *P* value <0.05 was considered statistically significant.

## Results

### Patient characteristics

**Figure 1.**
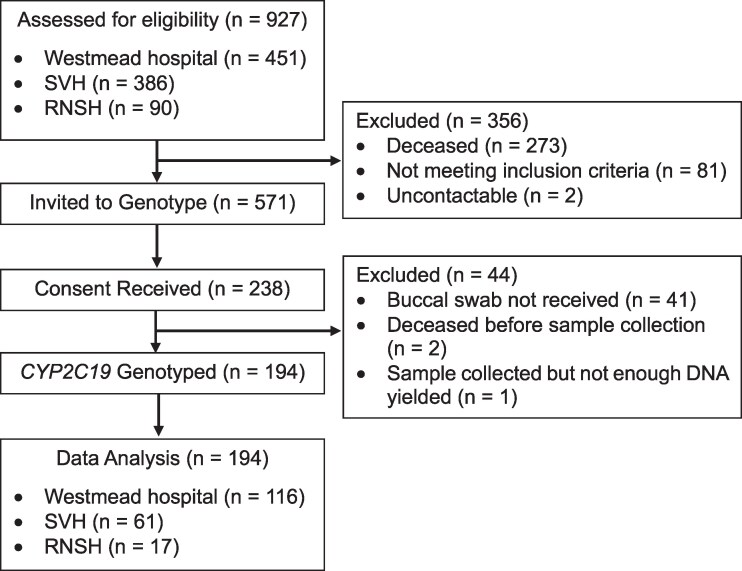
Recruitment flowchart. In total, 927 patients were assessed for eligibility to participate in the study across the three hospital sites and 194 were included in data analysis. RNSH, Royal North Shore Hospital; SVH, St Vincent’s Hospital.

A total of 194 patients were included (Figure 1) of whom 55% were male. The median age was 58 years, and 47% identified as Caucasian. Most patients were either *CYP2C19* intermediate (36%; 69/194) or normal (34%; 65/194) metabolizers, followed by rapid (21%, 41/194), ultrarapid (7%; 14/194) and poor (3%; 5/194) metabolizers (Table 2).

**Table 2.** Patient characteristics in the switched and non-switched group

Characteristic	Total (*n* = 194)	Switched (*n* = 62)	Non-switched (*n* = 132)	*P*
Age, y	58 (41–64)	61 (36–64)	57 (43–64)	0.7712
BMI^a^				0.4045
Underweight (<18.5 kg/m^2^)	10 (5%)	3 (5%)	7 (5%)	
Healthy (18.5–25 kg/m^2^)	77 (40%)	23 (37%)	54 (41%)	
Overweight (25–30 kg/m^2^)	60 (31%)	23 (37%)	37 (28%)	
Obese class I (30–35 kg/m^2^)	32 (17%)	6 (10%)	26 (20%)	
Obese class II (35–40 kg/m^2^)	11 (6%)	5 (8%)	6 (5%)	
Obese class III (>40 kg/m^2^)	2 (1%)	1 (2%)	1 (1%)	
Sex				0.1077
Male	107 (55%)	29 (47%)	78 (59%)	
Female	87 (45%)	33 (53%)	54 (41%)	
Ethnicity^b^				0.7128
European	91 (47%)	27 (44%)	64 (48%)	
Oceanian	67 (35%)	23 (37%)	44 (33%)	
Asian	31 (16%)	8 (13%)	23 (17%)	
African	6 (3%)	3 (5%)	3 (2%)	
Middle Eastern	8 (4%)	3 (5%)	5 (4%)	
Medical condition				0.8206
Haematological malignancy	125 (64%)	43 (69%)	82 (62%)	
Other malignancy	3 (2%)	1 (2%)	2 (2%)	
Heart transplant	30 (15%)	10 (16%)	20 (15%)	
Lung transplant	8 (4%)	1 (2%)	7 (5%)	
Renal transplant	2 (1%)	1 (2%)	1 (1%)	
Aplastic anaemia	4 (2%)	1 (2%)	3 (2%)	
Pneumonia	4 (2%)	2 (3%)	2 (2%)	
Other^c^	18 (9%)	4 (6%)	14 (11%)	
*CYP2C19* genotype				0.9197
*1/*1	65 (34%)	22 (35%)	43 (33%)	
*17/*17	14 (7%)	4 (6%)	10 (8%)	
*1/*17	41 (21%)	15 (24%)	26 (20%)	
*1/*2	48 (25%)	13 (21%)	35 (27%)	
*1/*3	4 (2%)	2 (3%)	2 (2%)	
*2/*17	17 (9%)	5 (8%)	12 (9%)	
*2/*2	5 (3%)	1 (2%)	4 (3%)	
*CYP2C19* phenotype				0.9041
Normal metabolizer	65 (34%)	22 (35%)	43 (33%)	
Ultrarapid metabolizer	14 (7%)	4 (6%)	10 (8%)	
Rapid metabolizer	41 (21%)	15 (24%)	26 (20%)	
Intermediate metabolizer	69 (36%)	20 (32%)	49 (37%)	
Poor metabolizer	5 (3%)	1 (2%)	4 (3%)	

Data presented as median (IQR) or *n* (% of column total).

^a^BMI missing for two patients.

^b^Ethnicity missing for 11 patients. Numbers greater than total *n* because some patients identified multiple ethnicities.

^c^Other, such as surgery/wounds, bronchiolitis, bronchiectasis, eye infections.

Voriconazole was prescribed as prophylaxis in 57% (110/194) of patients, primarily following haematopoietic stem cell or lung transplantation. Empirical therapy for suspected fungal infection (no organism identified) was initiated in 20% (39/194) of patients, while 14% (28/194) had probable or proven invasive aspergillosis. Non-invasive aspergillosis was documented in 1% (2/194) of patients, and 8% (15/194) had infections caused by non-*Aspergillus* fungi. Eight of these patients were infected with resistant *Lomentospora/Scedosporium* spp.

### CYP2C19 phenotype and switching antifungals

**Figure 2.**
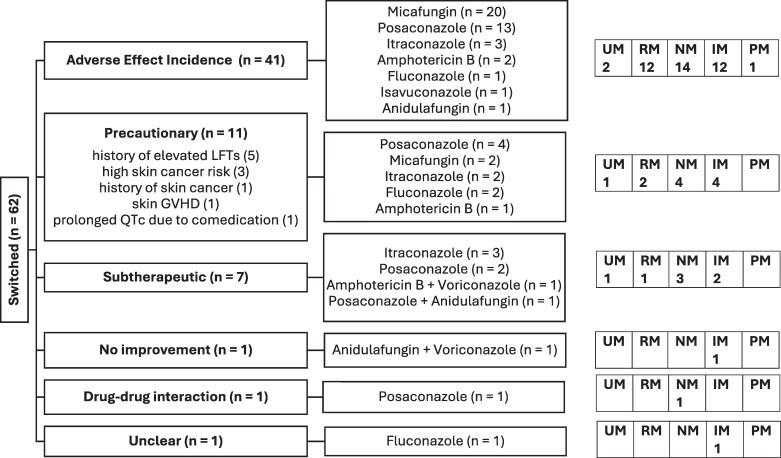
Flowchart depicting reason for switching from voriconazole therapy in the switched group. For each reason of cessation, the alternative antifungal used and *CYP2C19* phenotype of the patients were identified. A precautionary switch refers to switching due to reasons that were not the direct result of voriconazole therapy. ‘Precautionary’ reasons for switching were specified. For one of the patients taking voriconazole as prophylaxis, the reason for switching was unclear from the medical records. See Figure S1 for reasons for switching stratified by voriconazole indication. GVHD, graft-versus-host disease; IM, intermediate metabolizers; LFT, liver function test; NM, normal metabolizers; PM, poor metabolizers; RM, rapid metabolizers; UM, ultrarapid metabolizers.

One-third of patients (32%; 62/194) were switched from voriconazole monotherapy to either an alternative antifungal (97%; 60/62) or combination therapy (3%; 2/62). Among those switched, 35% (22/62) were normal and 32% (20/62) were intermediate metabolizers. Drug-related adverse effects were the most common reason for switching [66%; 41/62; see Figure 2 and Figure S1 (available as Supplementary data at *JAC* Online) for reasons stratified by indication]. Only seven patients (11%; 7/62) were switched due to subtherapeutic voriconazole concentrations. There was no association between *CYP2C19* phenotype and the requirement to switch antifungal therapy (*P* = 0.9041; Table 2; Table S1 for logistic regression).

#### Ultrarapid metabolizers—reasons for switching

There were 14 *CYP2C19* ultrarapid metabolizers, of whom 4 (29%) were switched to alternative therapy. Three were switched during prophylaxis: two due to hepatotoxicity (elevated bilirubin and GGT), and one due to anticipated phototoxicity in a patient with skin graft-versus-host disease. The only patient switched for subtherapeutic exposure despite receiving 900 mg/day had proven invasive aspergillosis and had continued voriconazole for 4 months before switching.

#### Rapid metabolizers—reasons for switching

There were 41 *CYP2C19* rapid metabolizers, of whom 15 (37%) were switched to alternative therapy, predominantly (80%; 12/15) because of drug-related adverse effects, usually elevated liver enzymes. Two patients were switched as a precaution: one due to a drug–dug interaction causing QTc prolongation, and the other due to pre-existing derangements on liver function. The remaining patient, being treated for possible aspergillosis, was switched due to subtherapeutic voriconazole concentrations. Of the two patients with probable/proven invasive aspergillosis, one completed therapy and one was switched due to hepatotoxicity.

#### Normal metabolizers—reasons for switching

Just over half the *CYP2C19* normal metabolizers completed voriconazole therapy (54%; 35/65). One-third were switched to alternative therapy (34%; 22/65), mainly due to drug-related adverse effects (14/22). Three patients switched therapy due to subtherapeutic voriconazole concentrations, and five for precautionary reasons (skin cancer risk/chronic derangement in liver function) or drug interactions.

Among the 17 patients (26%) with probable/proven invasive aspergillosis, 9 were switched to alternative therapy. Reasons for switching included drug-related adverse effects (4/9), a precautionary measure (3/9) or subtherapeutic voriconazole concentrations (2/9). Of the patients switched due to a drug-related adverse effect, two had a photosensitivity reaction after months of therapy, one had visual disturbances after 1 week of therapy and the fourth had elevated liver enzymes after 1 week of therapy. Of note, this patient was receiving a high IV dose of voriconazole (800 mg/day).

#### Intermediate metabolizers—reasons for switching

Twenty *CYP2C19* intermediate metabolizers (29%; 20/69) were switched to alternative therapy. Of these, 12 (60%; 12/20) were switched due to drug-related adverse effects, including skin reactions (3/12), elevated liver enzymes (6/12) and hallucinations/vivid dreams (3/12). The remaining eight patients switched either as a precautionary measure (4/8), due to subtherapeutic voriconazole concentrations (2/8), disease progression (1/8) or for an unknown reason (1/8).

In patients with probable/proven invasive aspergillosis (12%; 8/69), three switched to alternative antifungal therapy; one because of phototoxicity after 4 months of therapy and the other two patients due to either subtherapeutic voriconazole concentrations or disease progression.

#### Poor metabolizers—reasons for switching

Only one of five poor metabolizers (20%) was switched to alternative antifungal therapy. The patient experienced both hallucinations and elevation of liver enzymes while on the standard 400 mg/day for a suspected fungal infection and was switched to micafungin. Two patients receiving lower voriconazole doses completed their course of voriconazole; one ceased voriconazole therapy 2 weeks after starting treatment because the fungus was identified and an alternative antifungal was more suitable (first line). The remaining poor metabolizer successfully completed prophylaxis without experiencing an adverse effect despite having supratherapeutic voriconazole concentrations. None of the poor metabolizers had a probable/proven invasive aspergillosis infection.

#### Other factors and switching

There were no differences in age, sex, BMI and early pathology data between the switched and non-switched groups (Table 2 and Table 3). However, differences were observed in the final pathology data, including all liver function tests, C-reactive protein and creatinine levels (Table 3). Elevated liver function tests were the most common reason for switching. Median C-reactive protein levels were markedly higher in the switched group (51.5 mg/L; IQR 8.5–132.8 mg/L) compared with the non-switched group (4.7 mg/L; IQR 4–27.8 mg/L; *P* < 0.001). Median creatinine levels were slightly lower in the switched group (72.5 µmol/L versus 86.5 µmol/L), although both remained within the normal range.

**Table 3.** Voriconazole therapy details in the switched and non-switched group

Voriconazole therapy details	Switched (*n* = 62)	Non-switched (*n* = 132)	*P*
Voriconazole indication			0.1120
Probable/proven invasive aspergillosis	14 (23%)	14 (11%)	
Aspergillosis (non-invasive)	0 (0%)	2 (2%)	
Non-aspergillus fungal infection	2 (3%)	13 (10%)	
Suspected fungal infection (no fungus identified)	11 (18%)	28 (21%)	
Prophylaxis	35 (56%)	75 (57%)	
Duration of therapy, d	27 (13–101)	75 (41–105)	<0.0001
Final route of administration			0.0037
IV	16 (26%)	12 (9%)	
Oral	46 (74%)	120 (91%)	
Final dose administered			0.3663
<400 mg/day	8 (13%)	16 (12%)	
400 mg/day	44 (71%)	83 (63%)	
>400 mg/day	10 (16%)	33 (25%)	
Trough voriconazole concentrations^a^			0.2546
Subtherapeutic (<1.0 mg/L)	24 (39%)	61 (46%)	
Therapeutic (1.0–5.5 mg/L)	34 (55%)	53 (40%)	
Supratherapeutic (>5.5 mg/L)	1 (2%)	2 (2%)	
Voriconazole-related adverse effects			<0.0001
Yes, adverse effect recorded	45 (73%)	37 (28%)	
No adverse effect recorded	17 (27%)	95 (72%)	
Liver function tests			
AST earliest (<36 U/L)^b^	23 (17–38)	22 (15–32)	0.1738
AST latest (<36 U/L)^b^	43 (23–96)	26 (20–38)	0.0005
ALT earliest (<51 U/L)^b^	33 (22–49)	24 (16–40)	0.0115
ALT latest (<51 U/L)^b^	38 (21–117)	24 (16–41)	0.0007
ALP earliest (30–110 U/L)^b^	75 (63–91)	72 (50–94)	0.2344
ALP latest (30–110 U/L)^b^	131 (89–246)	106 (76–150)	0.0444
GGT earliest (5–50 U/L)^b^	54 (28–106)	47 (27–74)	0.4565
GGT latest (5–50 U/L)^b^	210 (89–432)	79 (46–182)	0.0002
Bilirubin earliest (0–20 µmol/L)^b^	11 (8–18)	11 (7–16)	0.3905
Bilirubin latest (0–20 µmol/L)^b^	11(8–23)	8 (6–12)	<0.0001
Total protein earliest (60–80 g/L)^b^	58 (54–63)	58 (53–63)	0.9149
Total protein latest (60–80 g/L)^b^	60 (54–66)	65 (61–68)	0.0002
Albumin earliest (33–48 g/L)^b^	32 (27–35)	31 (27–34)	0.6851
Albumin latest (33–48 g/L)^b^	31 (25–37)	36 (32 -39)	<0.0001
Other pathology			
CRP earliest (<5 mg/L)^b^	26 (7–100)	35 (9–121)	0.5595
CRP latest (<5 mg/L)^b^	52 (9–133)	5 (4–28)	<0.0001
Creatinine earliest^c^	66 (53–91)	72 (59–101)	0.1677
Creatinine latest^c^	73 (57–95)	87 (68–109)	0.0047

CRP, C-reactive protein; ALP, alkaline phosphatase.

Data presented as median (IQR) or *n* (% of column total).

^a^Pharmacokinetic modelling was not possible for 19 patients due to missing administration time/date data.

^b^Normal range.

^c^Normal creatinine range is 60–110 µmol/L for males and 45–90 µmol/L for females.

### CYP2C19 phenotype and voriconazole exposure

**Figure 3.**
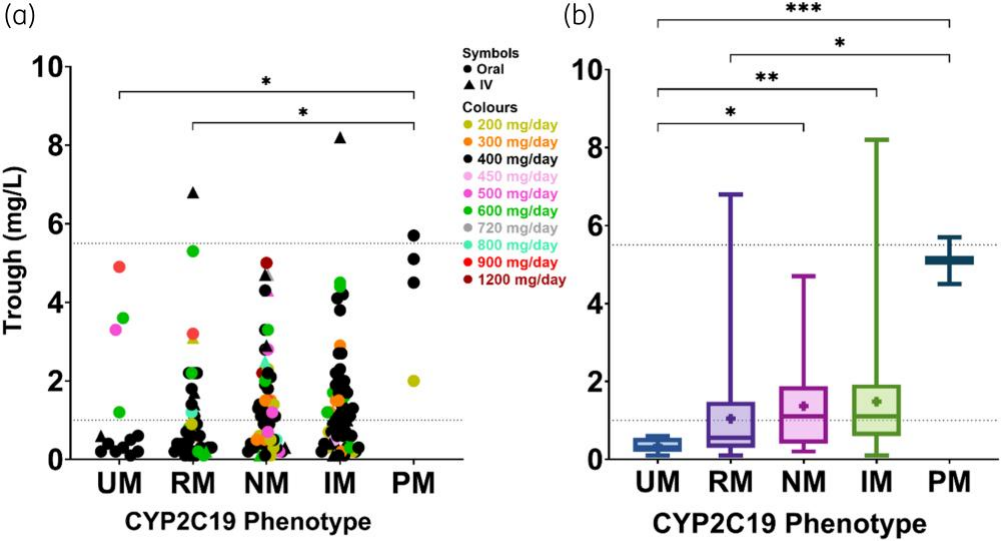
Relationship between *CYP2C19* phenotype and voriconazole trough concentrations. This includes average trough voriconazole concentrations for the last dosing regimen administered to all patients (*n* = 175) (a) and patients on voriconazole 400 mg/day (b). Trough voriconazole concentrations were measured as mg/L. All values were obtained from modelling the pharmacokinetic profile of patients using the Insight RX Nova software. For the scatter plot (a), the shapes represent the route of administration, and the colours represent the dose in mg/day. For the box-and-whisker plot (b), the boxplot presents the IQR with a horizontal band to signify the median and the whiskers representing the minimum and maximum values. The + sign represents the mean of each phenotype, and the dotted lines represent the upper and lower limits of the therapeutic range. Kruskal–Wallis test was conducted with correction for multiple comparisons using Dunn’s multiple comparisons test (*P < 0.05; **P < 0.01; ***P < 0.001; ****P < 0.0001). See Figure S2 for dose versus trough concentration graph. IM, intermediate metabolizers; NM, normal metabolizers; PM, poor metabolizers; RM, rapid metabolizers; UM, ultrarapid metabolizers. Figure generated in GraphPad Prism.

Pharmacokinetic modelling was performed for 175 of 194 patients; the remaining 19 patients were not modelled due to missing voriconazole administration time/date data (1 ultrarapid, 2 rapid, 9 normal, 6 intermediate and 1 poor metabolizer). Figure 3 shows the predicted average trough concentrations stratified by *CYP2C19* phenotype, dose and route of administration (Figure S2 for dose–exposure plot).

All ultrarapid metabolizers receiving 400 mg/day (69%; 9/13) had subtherapeutic trough voriconazole concentrations (range 0.1–0.6 mg/L). Therapeutic trough concentrations were attained only in ultrarapid metabolizers receiving ≥500 mg/day (31%; 4/13: 500 mg/day = 3.3 mg/L, 600 mg/day = 3.6 mg/L, 600 mg/day = 1.2 mg/L, 900 mg/day = 4.9 mg/L). Most rapid metabolizers (64%; 25/39), close to half the normal metabolizers (45%; 25/56) and a minority of intermediate metabolizers (41%; 26/63) had subtherapeutic trough voriconazole concentrations. Poor metabolizers typically had trough concentrations near the upper limit of the normal range (5.7, 5.1, 4.5 and 2.0 mg/L). The only poor metabolizer with a mid-range concentration (2.0 mg/L) was receiving 200 mg/day. Supratherapeutic concentrations were observed in three patients who were either poor metabolizers (1/3) or receiving IV therapy (2/3; one rapid and one intermediate metabolizer).

There were differences in trough voriconazole concentrations across *CYP2C19* phenotypes (Kruskal–Wallis statistic = 12.88, *P* = 0.0119). Specifically, trough voriconazole concentrations were lower in ultrarapid (median 0.5, IQR 0.2–2.3 mg/L) and rapid metabolizers (median 0.7, IQR 0.3–1.7 mg/L) compared with poor metabolizers (median 4.8, IQR 2.6–5.6 mg/L; *P* = 0.0172, *P* = 0.0141, respectively). Similar associations were found with AUC_0–24_ values (Figure S3). A comparison between patients receiving the 400 mg/day dose is shown in Table 4 (Table S2 for AUC_0–24_ values).

**Table 4.** *CYP2C19* phenotype versus trough voriconazole concentrations in patients on 400 mg/day (*n* = 116)

Troughs (median, IQR)	UM	RM	NM	IM	PM
UM (median 0.3, IQR 0.2–0.6 mg/L)	—	—	—	—	—
RM (median 0.6, IQR 0.3–1.5 mg/L)	0.7002	—	—	—	—
NM (median 1.1, IQR 0.4–1.9 mg/L)	**0**.**0257**	0.9785	—	—	—
IM (median 1.1, IQR 0.6–1.9 mg/L)	**0**.**0089**	0.3764	>0.9999	—	—
PM (median 5.1, IQR 4.5–5.7 mg/L)	**0**.**0008**	**0.0154**	0.1332	0.1706	—

IM, intermediate metabolizers; NM, normal metabolizers; PM, poor metabolizers; RM, rapid metabolizers; UM, ultrarapid metabolizers.

Data presented as *P* values from Dunn’s multiple comparisons test. Significant comparisons (*P* < 0.05) are bolded. Kruskal–Wallis test was significant for trough concentrations versus *CYP2C19* phenotype (Kruskal–Wallis statistic = 21.93, *P* = 0.0002).

### CYP2C19 phenotype and voriconazole-related adverse effects

**Figure 4.**
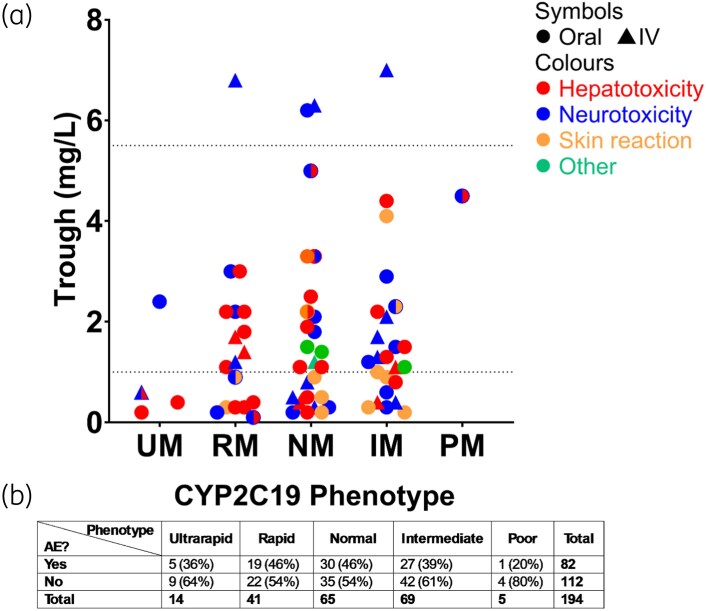
Influence of voriconazole trough concentrations and *CYP2C19* phenotype on the incidence of adverse effects. Average trough voriconazole concentrations were measured as mg/L. All values were obtained from modelling the pharmacokinetic profile of patients using the Insight RX Nova software. For the scatter plot (a), the shapes represent the route of administration, and the colours represent the type of adverse effect experienced. The dotted lines represent the upper and lower limits of the therapeutic range. Kruskal–Wallis test was conducted with correction for multiple comparisons using Dunn’s multiple comparisons test. (b) Table displaying the incidence of adverse effects across phenotypes. Fisher’s exact test was conducted and no statistically significant associations were identified (*P* < 0.05). See Figure S4 for influence of AUC_0–24_ values and *CYP2C19* phenotype on the incidence of adverse effects. AE, adverse event; IM, intermediate metabolizers; NM, normal metabolizers; PM, poor metabolizers; RM, rapid metabolizers; UM, ultrarapid metabolizers. Figure generated in GraphPad Prism.

Overall, 82 patients (42%; 82/194) experienced a voriconazole-related adverse effect. Rates were similar across *CYP2C19* phenotypes; 46% in both normal (30/65) and rapid (19/41) metabolizers, 39% in intermediate (27/69), 36% in ultrarapid (5/14) and 20% (1/5) in poor metabolizers. No statistically significant association between *CYP2C19* phenotype and adverse effect incidence (*P* = 0.7294; Figure 4; Figure S4) was identified.

#### Type of adverse effect

The most common drug-related adverse effects were hepatotoxicity (45%; 37/82) and neurotoxicity (44%; 36/82), followed by dermatological reactions including phototoxicity (17%; 14/82) and other effects (10%; 8/82), such as QTc prolongation, nausea or myalgia. A subset of patients (18%; 15/82) experienced multiple adverse effects. No relationship was observed between *CYP2C19* phenotype and the type of adverse effect (*P* = 0.9092; Table 5).

**Table 5.** *CYP2C19* phenotype versus type of voriconazole-related adverse effect recorded

		Phenotype					
		Ultrarapid *n* = 14	Rapid *n* = 41	Normal *n* = 65	Intermediate *n* = 69	Poor *n* = 5	Total
Type of AE	Hepatotoxicity	3 (21%)	11 (27%)	12 (18%)	10 (14%)	1 (20%)	37
	Neurotoxicity	3 (21%)	8 (20%)	13 (20%)	11 (16%)	1 (20%)	36
	Skin reaction	0 (0%)	2 (5%)	5 (8%)	7 (10%)	0 (0%)	14
	Other	0 (0%)	1 (2%)	5 (8%)	2 (3%)	0 (0%)	8
	Total	6	22	35	30	2	95

AE, adverse event.

Data presented as *n* (% of column total).

Neurotoxicity was observed in all patients with adverse effects and supratherapeutic trough voriconazole concentrations (*n* = 4). Additionally, neurotoxicity was common in patients receiving IV voriconazole, independent of drug exposure. Hepatotoxicity occurred across all phenotypes, drug exposure ranges and at different times throughout a course of therapy (Figure 4; Figure S5). Skin reactions and other non-hepatic effects were not associated with trough concentrations or *CYP2C19* phenotype. Notably, patients who experienced skin-related toxicity, muscle pain or QTc prolongation were often on long-term voriconazole therapy (>2 months; Figure S5).

## Discussion

In this multicentre, retrospective observational study, we investigated whether *CYP2C19* phenotype was associated with switching from voriconazole to alternative antifungal therapy, and explored relationships between *CYP2C19* phenotype, predicted voriconazole exposure and voriconazole-related adverse effects. Consistent with published data,^33^ we observed substantial variability in voriconazole exposure across *CYP2C19* phenotypes. Ultrarapid and rapid metabolizers in this study had significantly lower trough concentrations and AUC_0–24_ values than poor metabolizers, supporting the role of *CYP2C19* in influencing voriconazole clearance. Our results indicate no observed association between *CYP2C19* phenotype and the likelihood of switching antifungal therapy from voriconazole. Voriconazole-related adverse effects largely drove switching but did not differ clearly by *CYP2C19* phenotype. Importantly, antifungal prescribing decisions were made without access to pharmacogenomic information.

Our experience suggests that voriconazole prescribing in real-world practice is less influenced by the dose–response relationship than expected. Rather, it is influenced by a multitude of factors such as adverse effects and toxicity concerns, institutional practice, voriconazole concentrations, clinical deterioration, drug–drug interactions and diagnostic uncertainty. We designed this study to determine whether patients who ultimately required a change in therapy could be retrospectively linked to their *CYP2C19* phenotype, with the broader goal of understanding whether pharmacogenomics could support earlier dosing decisions and antifungal selection. We hypothesized that ultrarapid and rapid metabolizers would demonstrate higher switching rates due to underexposure, while poor metabolizers would switch due to toxicity. However, no relationship between *CYP2C19* phenotype and switching was observed. This negative finding does not diminish the potential value of pharmacogenomic testing but highlights that switching in this patient group is a complex and subjective clinical decision and may not be an ideal endpoint for the evaluation of the utility of pharmacogenomics in antifungal prescribing decisions.

Nevertheless, a few other factors need to be considered when interpreting our findings. Firstly, given only a small number of patients were poor metabolizers, the study was underpowered to detect phenotype-specific differences in switching and toxicity in this group. Secondly, given that significantly higher C-reactive protein levels were observed in patients who were switched to alternative therapy, inflammation-mediated suppression of *CYP2C19* activity could have obscured the genotype-phenotype relationship.^27^ Inflammation is known to have an inhibitory effect on *CYP2C19*, with a 50 mg/L increase in C-reactive protein associated with a 35% increase in voriconazole trough concentration.^34^ Finally, given that therapeutic drug monitoring (TDM) was routinely used within all three tertiary hospitals, and two of the hospitals had antimicrobial stewardship programmes, there may have been a reliance on those avenues to guide dosing. This may have contributed to the limited switching of therapy due to subtherapeutic concentrations.

It is notable, however, that although TDM was used across the participating hospitals, subtherapeutic voriconazole trough concentrations were still common. This disconnect may reflect limitations in TDM implementation. In Australian centres, delays in sampling and turnaround times,^35^ combined with variable clinician experience in TDM interpretation, are well documented.^36^ It has been previously shown that accurate TDM sampling and interpretation significantly reduce rates of voriconazole discontinuation due to adverse events and lead to significantly higher rates of complete or partial response to voriconazole therapy (81% in TDM group versus 57% in non-TDM group).^8^ Our study also shows the potential impact of TDM when applied appropriately. We assessed exposure based on the patients’ final dosing regimens. Therefore, where TDM was applied, we were able to capture TDM-adjusted regimens. Interestingly, all ultrarapid metabolizers on 400 mg/day had subtherapeutic voriconazole trough concentrations, and only ultrarapid metabolizers on high doses of voriconazole were within the therapeutic range. Similarly, only the poor metabolizer on a low dose of voriconazole was well within the therapeutic range. This finding highlights an important implication for practice, namely that while TDM-guided dose adjustment can eventually address inadequate voriconazole exposure, reliance on TDM alone means that opportunities for early optimization of therapy may be missed.

Pharmacogenomic testing, if conducted early, could help triage patients’ dose requirements, which can subsequently be finessed using TDM. Patients who demonstrate voriconazole exposure extremes and who are unlikely to respond to standard dosing could be identified early. Clinicians and pharmacists would be able to more confidently prescribe or recommend, respectively, atypical doses of voriconazole or alternative antifungal therapy. Theoretically, this could help prevent futile dose escalations and improve treatment outcomes, especially in patients with invasive aspergillosis where achievement of therapeutic antifungal concentrations is time critical. Indeed, previous work by Patel *et al.*^37^ demonstrated that rapid/ultrarapid metabolizers initiated on a phenotype-guided 600 mg/day were less likely to have subtherapeutic concentrations at the first steady-state measurement than those started on standard dosing (16% versus 70%; *P* < 0.001). Logistically, given that stem cell and solid organ transplant recipients are at high risk of infection with invasive aspergillosis, pre-emptive pharmacogenomic testing could be implemented during transplantation work-up.

The study’s strengths include its multicentre design, incorporation of pharmacokinetic modelling to determine true trough voriconazole concentrations with AUC_0–24_ values, and the detailed review of clinical decision-making. It also represents one of the largest real-world cohorts to examine *CYP2C19* phenotype alongside antifungal switching patterns. However, its retrospective design introduces selection bias as only patients who were alive and contactable could participate. Therefore, the study cohort represents a subset of survivors; patients who had invasive aspergillosis and subsequently died were not represented. Additionally, had the test been integrated into routine clinical practice, the outcomes and interpretations might have differed.

In summary, our findings suggest that while *CYP2C19* phenotype correlates with voriconazole exposure, it does not reliably predict antifungal treatment modification in practice, a complex multifactorial decision. Our observations on voriconazole dosing and exposure support the potential complementary use of *CYP2C19* genotyping alongside TDM. Pre-emptive *CYP2C19* genotyping can help identify patients who require non-standard dosing of voriconazole and facilitate selection of the most appropriate starting dose or alternative antifungal therapy. Thereafter, TDM can be used to guide timely dosing adjustments to increase target attainment. Future steps should include the routine monitoring of serum galactomannan as a response biomarker to further personalize treatment.^38^ We anticipate that ongoing randomized controlled trials of *CYP2C19* genotype-guided voriconazole prescribing will focus on poor and ultrarapid metabolizers and include cost-effectiveness analyses to guide real-world implementation.^39^

## Supplementary Material

dkag168_Supplementary_Data

## Supplementary data

Figures S1 to S5 and Tables S1 and S2 are available as Supplementary data at *JAC* Online.
