# Supplementary material for: Voriconazole therapy and CYP2C19 phenotype: identifying patients who may need alternative antifungal therapy
Source: J Antimicrob Chemother. 2026 May 19;81(6):dkag168. doi: 10.1093/jac/dkag168 (PMC13184621; doi:10.1093/jac/dkag168)
Supplement: dkag168_Supplementary_Data [file dkag168_supplementary_data.docx]

| **UM** | **RM**  **1** | **NM**  **4** |
| --- | --- | --- |
| **IM**  1 | **PM** |  |

| **UM**  **2** | **RM**  **8** | **NM**  **7** |
| --- | --- | --- |
| **IM**  **9** | **PM** |  |

**Proven or probable invasive aspergillosis (n = 28)**

**Prophylaxis (n = 110)**

| **UM** | **RM** | **NM**  **1** |
| --- | --- | --- |
| **IM**  1 | **PM** |  |

| **UM**  **1** | **RM** | **NM**  **2** |
| --- | --- | --- |
| **IM**  1 | **PM** |  |

| **UM** | **RM** | **NM** |
| --- | --- | --- |
| **IM**  1 | **PM** |  |

| **UM**  **1** | **RM**  **2** | **NM**  **1** |
| --- | --- | --- |
| **IM**  **2** | **PM** |  |

| **UM** | **RM** | **NM**  **3** |
| --- | --- | --- |
| **IM** | **PM** |  |

| **UM** | **RM** | **NM** |
| --- | --- | --- |
| **IM**  1 | **PM** |  |

**Fungal infection - not invasive aspergillosis (n = 17)**

**Suspected fungal infection (n = 39)**

| **UM** | **RM**  **1** | **NM** |
| --- | --- | --- |
| **IM** | **PM** |  |

| **UM** | **RM**  **2** | **NM**  **3** |
| --- | --- | --- |
| **IM**  **2** | **PM**  **1** |  |

| **UM** | **RM**  **1** | **NM** |
| --- | --- | --- |
| **IM** | **PM** |  |

| **UM** | **RM** | **NM**  **1** |
| --- | --- | --- |
| **IM** | **PM** |  |

| **UM** | **RM** | **NM** |
| --- | --- | --- |
| **IM**  2 | **PM** |  |

**Supplementary Figure 1. Reason for switching from voriconazole therapy in the switched group stratified by voriconazole indication.** This is presented as a flowchart and there are four indications (proven/probable invasive aspergillosis; fungal infection but not invasive aspergillosis; suspected fungal infection; prophylaxis). For each reason of cessation, alternative antifungal used and *CYP2C19* phenotype of patients were identified. A precautionary switch refers to switching due to reasons that were not the direct result of voriconazole therapy. “Precautionary” reasons for switching were specified.

**
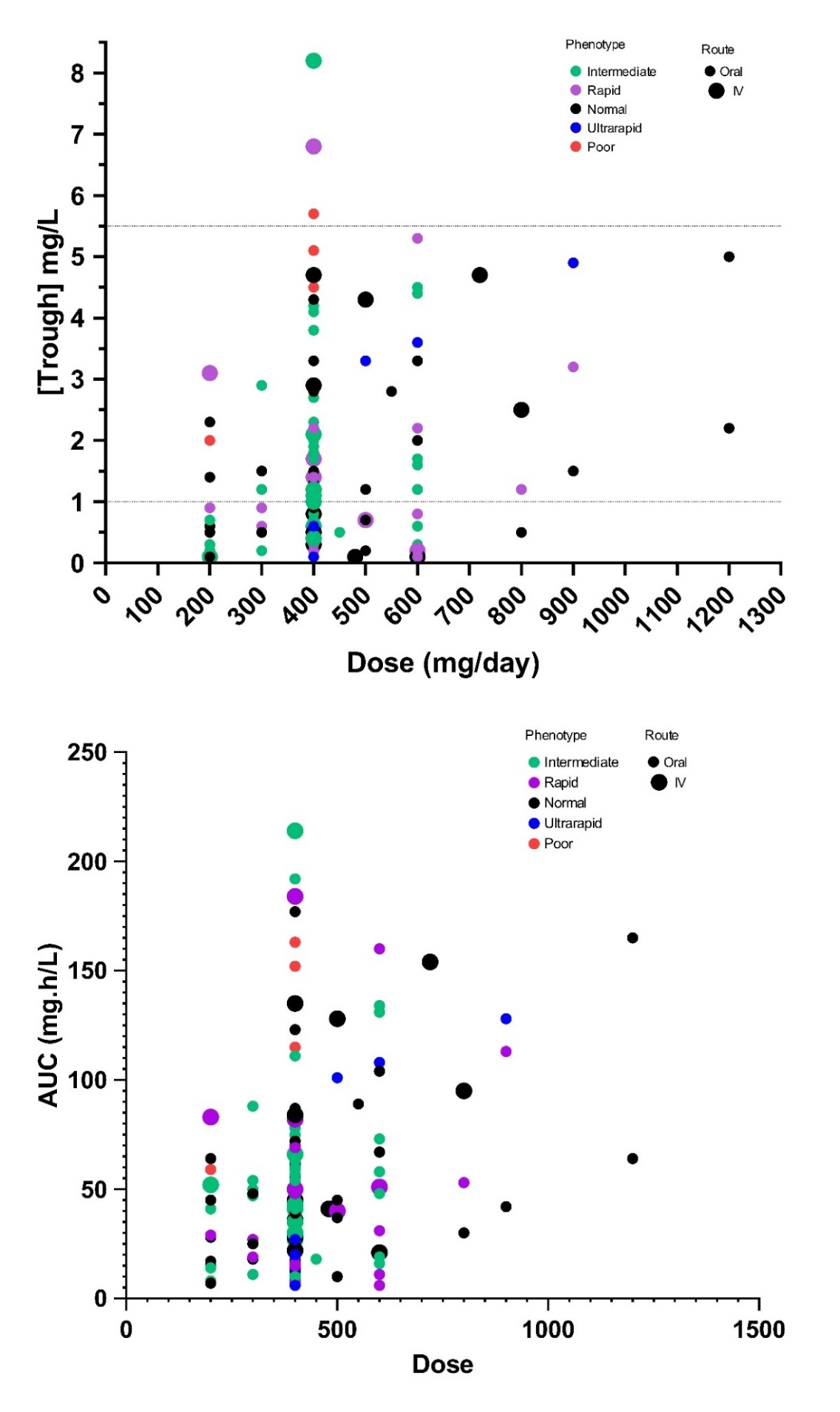
**

**Supplementary Figure 2. Dose-exposure plot stratified by *CYP2C19* phenotype and route of administration.** Trough concentrations were measure as mg/L and dose as mg/day. All values were obtained from modelling the pharmacokinetic profile of patients using the Insight RX Nova software. For the scatter plot, the size represents the route of administration, and the colours represent the phenotypes. The dotted line represents the upper and lower limit of the therapeutic range. Abbreviations: IV, intravenous. Figure generated in GraphPad Prism.

**
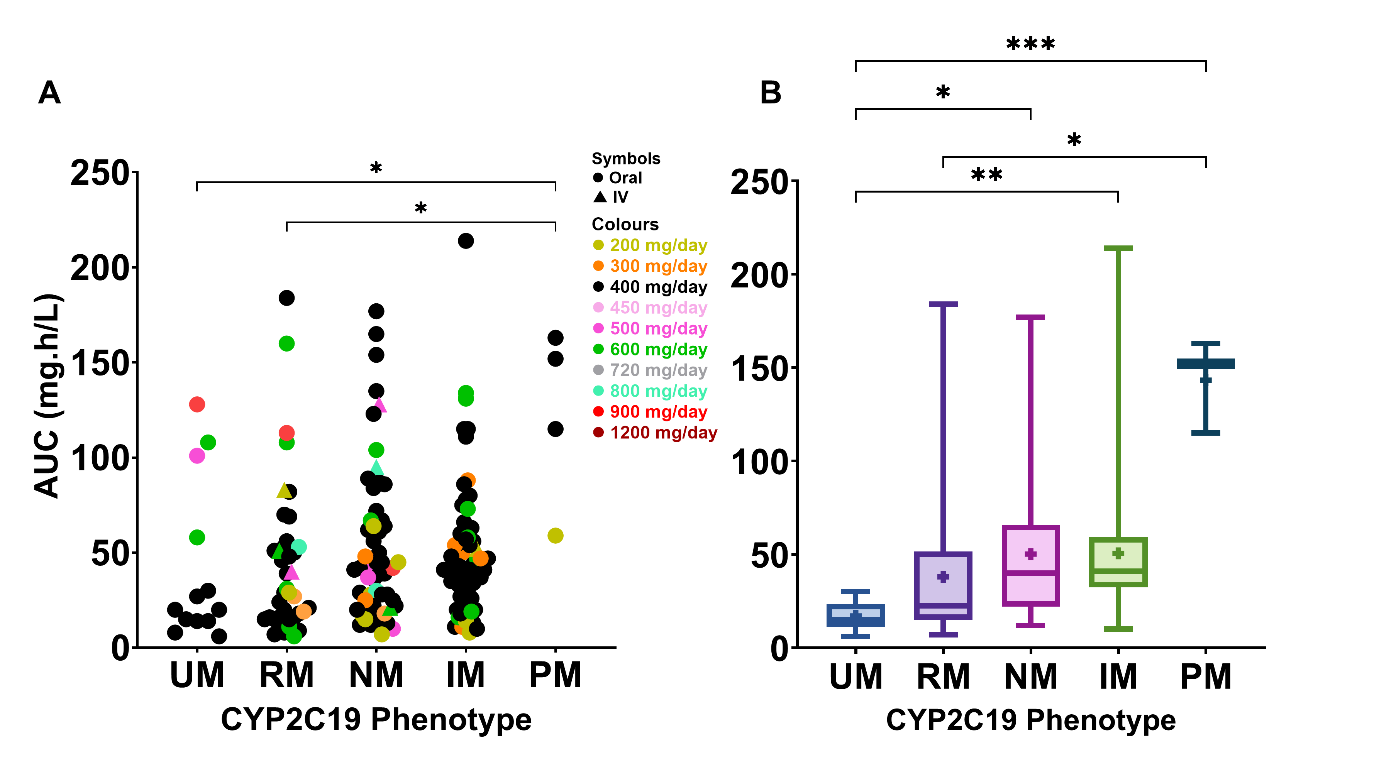
Supplementary Figure 3. The relationship between *CYP2C19* phenotype and voriconazole exposure.** This includes average AUC_0-24_ values for the last dosing regimen administered to all patients (n = 175) (A) and patients on voriconazole 400 mg/day (B). AUC_0-24_ values were measured as mg.h/L. All values were obtained from modelling the pharmacokinetic profile of patients using the Insight RX Nova software. For the scatter plot (A), the shapes represent the route of administration, and the colours represent the dose in mg/day. For the box-and-whisker plot (B), the boxplot presents the interquartile range with a horizontal band to signify the median and the whiskers represent the minimum and maximum values. The + sign represents the mean of each phenotype. Kruskal-Wallis test was conducted with correction for multiple comparisons using Dunn’s multiple comparisons test (*p<0.05; **p<0.01; ***p<0.001; ****p<0.0001). Abbreviations: UM, ultrarapid metabolisers; RM, rapid metabolisers; NM, normal metabolisers; IM, intermediate metabolisers; PM, poor metabolisers; IV, intravenous. Figure generated in GraphPad Prism.

**
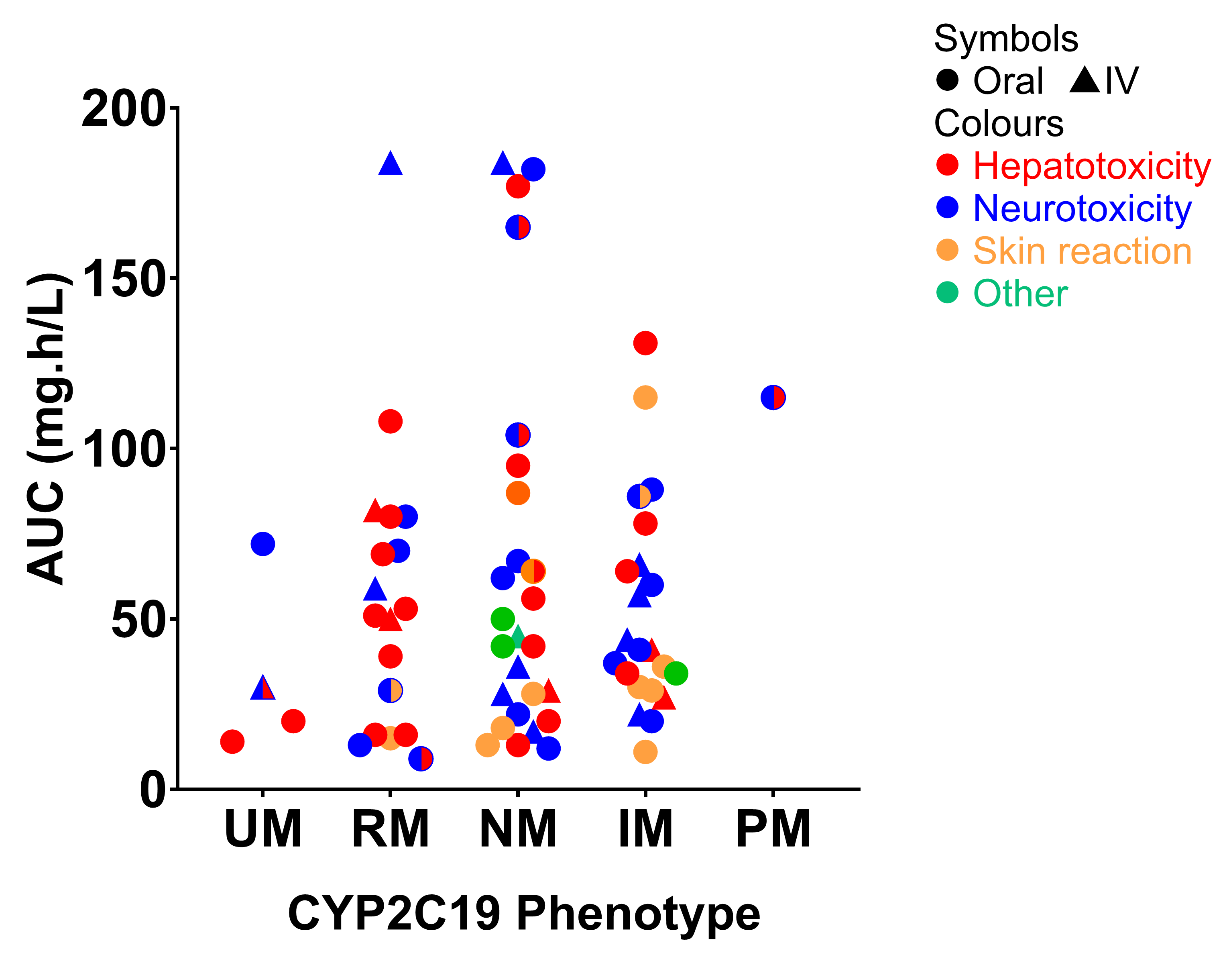
**

**Supplementary Figure 4. Influence of AUC_0-24_ values and *CYP2C19* phenotype on the incidence of adverse effects.** AUC_0-24_ values were measured as mg/L. All values were obtained from modelling the pharmacokinetic profile of patients using the Insight RX Nova software. For the scatter plot, the shapes represent the route of administration, and the colours represent the type of adverse effect experienced. Kruskal-Wallis test was conducted with correction for multiple comparisons using Dunn’s multiple comparisons test. No statistically significant associations were identified (p<0.05). Abbreviations: UM, ultrarapid metabolisers; RM, rapid metabolisers; NM, normal metabolisers; IM, intermediate metabolisers; PM, poor metabolisers; IV, intravenous. Figure generated in GraphPad Prism.


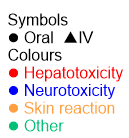

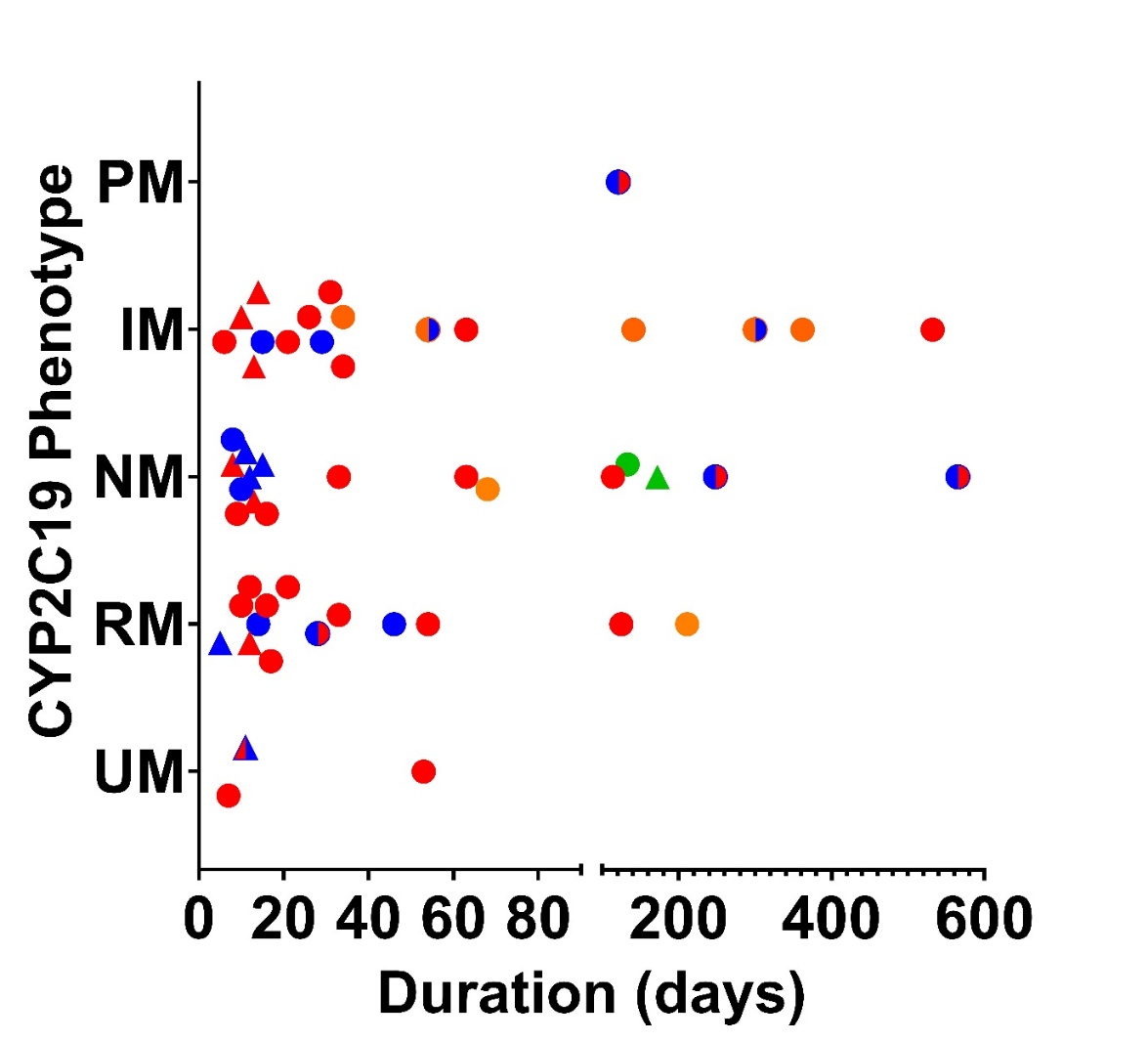


**Supplementary Figure 5. Time to onset of adverse effects leading to voriconazole therapy discontinuation, stratified by phenotype.** Time was measured as days. For the scatter plot, the shapes represent the route of administration, and the colours represent the type of adverse effect experienced. Abbreviations: UM, ultrarapid metabolisers; RM, rapid metabolisers; NM, normal metabolisers; IM, intermediate metabolisers; PM, poor metabolisers; IV, intravenous. Figure generated in GraphPad Prism.

**Supplementary Table 1. Multiple logistic regression output.**

|  | **Switching** | | |
| --- | --- | --- | --- |
|  | **OR** | **95% confidence interval** | **p-value** |
| **Ultrarapid metabolisers** | 0.7115 | 0.03384 to 5.607 | 0.7740 |
| **Rapid metabolisers** | 1.1360 | 0.1826 to 5.8200 | 0.8821 |
| **Normal metabolisers (reference)** | - | - | - |
| **Intermediate metabolisers** | 0.8196 | 0.2308 to 2.7860 | 0.7513 |
| **Poor metabolisers** | 0.6912 | 0.02684 to 9.682 | 0.7880 |

OR: Odds ratio, adjusted for latest C-reactive protein, aspartate aminotransferase, alanine aminotransferase, alkaline phosphatase, gamma-glutamyl transferase and bilirubin levels.

| **AUC_0-24_ (median, IQR)** | **UM** | **RM** | **NM** | **IM** | **PM** |
| --- | --- | --- | --- | --- | --- |
| **UM** (median 15.0, IQR 11.0-23.5 mg.h/L) | **-** | **-** | **-** | **-** | **-** |
| **RM** (median 22.5, IQR 15.0-51.5 mg.h/L | 0.4332 | **-** | **-** | **-** | **-** |
| **NM** (median 40.0, IQR 22.0-65.8 mg.h/L) | **0.0145** | >0.9999 | **-** | **-** | **-** |
| **IM** (median 41.0, IQR 32.3-59.3 mg.h/L) | **0.0031** | 0.2934 | >0.9999 | **-** | **-** |
| **PM** (median 152.0, IQR 115.0-163.0 mg.h/L) | **0.0007** | **0.0208** | 0.1579 | 0.2425 | **-** |

**Supplementary Table 2. *CYP2C19* phenotype versus AUC_0-24_ in patients on 400 mg/day (n = 116)**

Data presented as p values from Dunn’s multiple comparisons test. Significant comparisons (p < 0.05) are bolded. Kruskal-Wallis test was significant for AUC_0-24_ vs *CYP2C19* phenotype (Kruskal-Wallis statistic = 23.29, p = 0.0001). Abbreviations: UM, ultrarapid metabolisers; RM, rapid metabolisers; NM, normal metabolisers; IM, intermediate metabolisers; PM, poor metabolisers; IQR, interquartile range.
